# An Epi‐Metabolomics Approach for Studying Epigenetic and Metabolic changes in Alzheimer's Disease Brain

**DOI:** 10.1002/alz70856_101070

**Published:** 2025-12-25

**Authors:** Nadia Ashrafi, Sangeetha Vishweswaraiah, Ali Yilmaz, Sumeyya Akyol, Ieva Kerševičiūtė, Karolis Krinickis, Juozas Gordevičius, Stewart F Graham

**Affiliations:** ^1^ Corewell Health Research Institute, William Beaumont University Hospital, Royal Oak, MI, USA; ^2^ Corewell Health Research Institute, William Beaumont University Hospital, Oakland University‐William Beaumont School of Medicine, Royal Oak, MI, USA; ^3^ NX Prenatal Inc, Bellaire, TX, USA; ^4^ VUGENE, LLC, Grand Rapids, MI, USA

## Abstract

**Background:**

Alzheimer's Disease (AD) is a complex, multifactorial, progressive, and irreversible neurodegenerative disorder characterized by cognitive, functional, and behavioral impairments. The diagnosis of AD is based on the presence of amyloid plaques and intracellular neurofibrillary tangles, with pathological changes beginning 20 to 30 years before symptoms appear. Current treatments only slow disease progression and manage symptoms, while research remains focused on single omics approaches such as genomics, metabolomics, proteomics, and lipidomics, with the high cost of multi‐omics integration limiting deeper insight into its neuropathology. This study's novelty lies in integrating metabolomics and methylation analysis to investigate the etiology and pathogenesis of AD using post‐mortem brain samples from individuals with AD and mild‐AD, compared to age and gender‐matched controls.

**Method:**

A targeted LC‐MS/MS, ^1^H NMR and the Illumina Infinium Methylation EPIC Bead Chip assay, we identified differentially abundant metabolites and differentially methylated cytosines using robust linear regression. We further examined the correlation between methylation and metabolite in brain samples from individuals with AD (*n* = 30), mild‐AD (*n* = 14), and age and gender‐matched controls (*n* = 30).

**Result:**

20 metabolites were significantly different concentrations when we compared AD against controls (FDR q < 0.05). Similarly, 17 metabolites were identified as being at significantly different concentrations when we compared mild‐AD against controls (FDR q < 0.05). We identified 18 differentially methylated CpGs when comparing AD to controls and 48 CpGs when comparing Mild AD to control. Epimetabolome analysis corroborated our initial metabolomics analysis highlighting specific CpGs associated with the proteins of interested to be either hypo or hypermethylated. Inflammatory regulators, serotonergic synapse, and sphingolipid metabolism were all upregulated metabolic pathways in mild‐AD which could be directly linked to disease development. We also report significant perturbation in the biosynthesis of amino acids, 2‐Oxocarboxyclic acid metabolism, Starch, and sucrose metabolism those individuals who died from AD.

**Conclusion:**

Overall, our findings demonstrate intricate relationship between methylation changes and metabolite concentrations which underlines the utility of combining metabolomics and other omics‐based platforms such as epigenetics for the study of AD and related dementias.

